# The State of Autoregulation

**DOI:** 10.1007/s12028-020-01021-2

**Published:** 2020-06-16

**Authors:** Stefan Wolf

**Affiliations:** Department of Neurosurgery, Charité - Universitätsmedizin Berlin, Charitéplatz 1, 10117 Berlin, Germany

Cerebral autoregulation is a well-identified therapeutic target after traumatic brain injury (TBI) in current guidelines and recommendations [1–3]. It represents an independent predictor for worse outcome, besides demographic and injury severity factors known on admission [4]. Targeting cerebral autoregulation may facilitate to individualize treatment in terms of precision medicine [5]. One way suggested is to identify an individual threshold for lowering of intracranial pressure (ICP) [6, 7], another one to determine the optimal cerebral perfusion pressure (CPP) for a specific patient [8, 9]. Besides well-known pressure reactivity index (PRx), a myriad of ways to assess cerebral autoregulation has been described, a plethora which even the proponents of this research describe as daunting [10].

The work of Bennis et al., presented in this issue of Neurocritical Care, advances previous knowledge in several fresh ways [11]. The authors were looking to enhance 6-month outcome prediction from the CRASH II model (corticosteroid randomisation after significant head injury trial, [12]) by adding autoregulation data gathered in the first 24 h after start of neuromonitoring. Investigated were 45 moderately to severely injured TBI patients from two centers in the Netherlands. Logistic regression modeling was used with leave-one-out cross-validation and forward feature selection to get the best predictive capabilities. The favored model included the CRASH II score as fixed parameter and, being potentially amenable to treatment, mean arterial blood pressure (ABP) as well as various raw and processed cerebrovascular reactivity indices as selectable features. The paper is adding to the evidence that cerebrovascular reactivity/autoregulation is of importance after TBI. The statistical methods used are advanced, but well described and sound. Interpretation of findings and discussion are carefully worded to avoid overly enthusiastic interpretation.

Main asset of the work by Bennis et al. is the focus on the early hours after injury. Most other research, including the methods for individualizing treatment or the analysis of the imperative database with 1146 patients from Cambridge, UK, spanning a 25-year period [13], gather the whole time of monitoring in a single number reflecting “the” state of autoregulation. This resembles a pathologist’s view: knowing something went ugly, but unable to change that fate. However, if monitoring the state of autoregulation is to be of importance, numbers reflecting it are subject to changes over time. Patients stabilizing and surviving their initial primary injury will get improved mean monitoring values by a longer observation time alone. Therefore, the initial phase with the brain being highly vulnerable for secondary damage requires premium attention. Minor flaw #1: availability of advanced neuromonitoring required a specialist to set up the computer. Thus, analysis was performed for the first 24 h after availability of the specialist, not as soon as possible after injury, which would have been more compelling and may make a subtle difference. Minor flaw #2: the best predictive model identified included only the first 6 h after availability of the monitoring specialist instead of the whole period of 24 h… does this mean that less monitoring is more? We don’t know.

A second asset in the work of Bennis et al. is the use of appropriate machine learning methods as statistical tool [14]. Therefore, the results are likely to reflect best what is in the data: they are data driven and unlikely to be dependent on strong predefined assumptions. The only simple parameter present in every model was ABP. Lower ABP was related to a higher chance of unfavorable outcome. The reason why ICP was irrelevant remains unclear. My guess is that ICP was already well controlled (mean ~ 11.8 ± 5.6 mmHg, summarized from the paper’s supplementary Table 1 [11]) in a series of predominantly Marshall grade II patients. In contrast, mean ABP was ~ 81 ± 7 mmHg, measured at heart level in three quarters of the patients. This is rather on the low side of recommendations—if patients are nursed with 30° head of bed elevated, the resulting CPP may have well been below 60 mmHg in a considerable number.

Concerning the choice of relevant autoregulation parameters, the statistical models were volatile without clear preference. Impairment of PRx (defined as number of samples above a threshold of 0.35 divided by the total number of samples) was included among predictors for outcome, as well as the slope of the pressure amplitude index (PAx) (the 5-min Pearson correlation of ABP with the amplitude of ICP, and from this the change over the monitoring period assessed by linear regression). Such parameters are non-intuitive at the bedside and not easily accessible for intervention. It fits to the notion that, so far, no single therapeutic measure is known to restore a disturbed cerebral autoregulation after TBI [5]. This is in marked contrast to monitoring parameters like ICP or brain tissue oxygenation, where recommended treatment paradigms do exist [2, 3].

The volatility in autoregulation parameters found to be relevant is likely attributable to the small patient number. This calls for repeating the analysis in a larger multicentric dataset, e.g., the CENTER TBI high-resolution database. Promising candidates related to outcome may then get investigated as biomarkers whether different treatment paradigms like deeper or lighter sedation, choice of narcotic agent, use/nonuse of muscle relaxants, the level of hypocapnia vs strict normocapnia or strict vs liberal temperature control do have influence. It is well known that the yield on finding an optimal CPP by investigating PRx autoregulation may differ between centers [15] (being bicentric is another asset of the work of Bennis et al., although no differences between centers beyond the difference in ABP levels are given—minor flaw #3). This may lead to identify a less hostile environment in the intensive care unit for a severely injured TBI patient, with better control of secondary brain damage, and present a novel way in autoregulation research beyond being a mere prognostic biomarker or a tool to individualize treatment.

What is the current state of autoregulation research? It is certainly not as easy as the SIBICC recommendations suggest: “increase MAP by 10 mmHg and consider the state of autoregulation to get a lower ICP reading” [2]. As with every other non-trivial relationship, it may get complicated. Bennis et al. asked the question which parameter is of importance in the most vulnerable phase after TBI and tried to answer with appropriate methods. This approach may lead the way to a bright future of autoregulation research.
